# Highly Efficient Homozygous CRISPR/Cas9 Gene Editing Based on Single-Cell-Originated Somatic Embryogenesis in *Liriodendron tulipifera*

**DOI:** 10.3390/plants14030472

**Published:** 2025-02-05

**Authors:** Cairong Li, Pengshuo Jiang, Jiaji Zhang, Dingjie Yang, Lu Lu, Zhaodong Hao, Yingxuan Ma, Jisen Shi, Jinhui Chen

**Affiliations:** State Key Laboratory of Tree Genetics and Breeding, Co-Innovation Center for Sustainable Forestry in Southern China, Nanjing Forestry University, Nanjing 210037, China; licairong@njfu.edu.cn (C.L.); pengshuo77@gmail.com (P.J.); zhangjiaji0609@163.com (J.Z.); Yangdj@njfu.edu.cn (D.Y.); lulu2020@njfu.edu.cn (L.L.); haozd@njfu.edu.cn (Z.H.); yma@njfu.edu.cn (Y.M.)

**Keywords:** CRISPR/Cas9, *LtPDS*, *Liriodendron*, gene editing, mutation efficiency, embryogenic callus

## Abstract

The clustered, regularly interspaced short palindromic repeats (CRISPR)/CRISPR-associated protein (Cas) system is the most widely used gene-editing tool to date. However, its application in the genetic improvement of forestry trees has been largely limited. Here, we first established a highly efficient multi-target editing system in the magnoliid woody plant *Liriodendron tulipifera*. Using *phytoene desaturase* gene (*PDS*) as an example, we systematically compared CRISPR/Cas9 and CRSPR/Cpf1 expression systems for loss-of-function analysis and conducted genetic transformations using transient and stable transformation. Ultimately, our findings indicated that the CRISPR/Cas9 system, when applied to transformation based on single-cell-originated somatic embryogenesis, yielded the highest gene-editing efficiency, with mutation rates of nearly 100%. Furthermore, we obtained a total of 137 regeneration plantlets via somatic embryogenesis, of which 82.48% exhibited an albino phenotype. The Illumina sequencing results of albino seedlings and the callus tissue obtained from dedifferentiation of mutant plants revealed that the mutation at the T1 target site was homozygous. These results indicate that CRISPR/Cas9-based multiplex genome-editing technology can not only accelerate the identification of gene function but also be incorporated into the genetic improvement and breeding of tulip trees, supporting the scale propagation of genome-edited plantlets via somatic embryogenesis.

## 1. Introduction

Gene editing is a new genetic engineering technology that can modify specific points in an organism’s genome and has shown great potential in gene research, gene therapy, and genetic improvement. Currently, the most widely applied gene-editing technologies include meganucleases (MNs), zinc-finger nucleases (ZFNs), translation activator-like effector nucleases (TALENs), and clustered regularly interspaced short palindromic repeats/CRISPR-associated gene systems (CRISPR/Cas) [1]. Compared to the other three technologies, the CRISPR/Cas system is a more widely used gene-editing tool because of its simpler operation, relatively simple vector structure, and lower production cost [2]. Most importantly, the greatest advantage of CRISPR/Cas9 is that the Cas9 protein can effectively target multiple sites for gene editing under the guidance of multiple gRNAs [3,4].

*Liriodendron* is a large deciduous tree of the genus *Liriodendron* in Magnoliaceae. It is a relic plant of the Tertiary Period and is a unique and rare plant in China. There are only two species in nature, namely, *Liriodendron chinense* (Hemsl.) Sarg and *Liriodendron tulipifera* L. [5]. Classified as a national second-class rare and endangered protected plant, *Liriodendron* is not only valued for its superior timber quality but also cherished for its aesthetic appeal and ecological significance, making it an ideal choice for urban greening. As the demand for *Liriodendron* continues to grow, the species’ poor natural reproductive capacity and low natural fruit set rate have become critical issues. Traditional breeding methods are no longer sufficient to meet market demands. Therefore, research into breeding techniques that can shorten the breeding cycle and achieve rapid large-scale propagation are particularly important. In 2003, researchers such as Jinhui Chen, leveraging the theories of cell totipotency and molecular regulation of cell development, successfully established a somatic embryogenesis system for *Liriodendron* and cultivated somatic embryo-regenerant plants of *Liriodendron hybrid*, marking a breakthrough in somatic embryogenesis technology [6]. This achievement significantly propelled the large-scale production of *Liriodendron* and provided a new avenue for the development of *Liriodendron hybrid*.

The significance of somatic embryogenesis lies in its ability to enable plant cells to develop into complete plants without fertilization, which is revolutionary for rapid plant propagation and genetic improvement. Observations of the developmental processes of both zygotic and somatic embryos in *Sorbus pohuashanensis* have shown that the developmental pathways of somatic embryos are similar to those of zygotic embryos, both undergoing globular, heart-shaped, and torpedo-shaped stages before maturing into a full embryo. Research indicates that somatic embryos, whether they arise directly or indirectly on the surface of callus tissue, originate from single cells, providing cytological evidence for the single-cell origin of somatic embryos [7]. In the origin and developmental process of somatic embryos in Liriodendron hybrid, the results show that some single cells with a large nuclear–cytoplasmic ratio on the surface of the embryogenic callus develop into single-celled proembryos [8]. These single-celled proembryos undergo cell division to establish polarity and eventually develop into mature cotyledonary embryos, supporting the concept of the single-cell origin of somatic embryogenesis. Therefore, the single-cell-origin somatic embryogenesis system can reduce the chimeras produced by gene editing and is an important method for fixing beneficial somatic mutations. Somatic embryogenesis not only plays a significant role in plant propagation and genetic improvement but also provides an effective platform for gene-editing technology, making the targeted improvement of plant genes possible.

In 2018, Professor Jisen Shi’s team first deciphered the genome of *Liriodendron* [5]. This achievement is of great theoretical and practical significance for research on the evolution and genetics of *Liriodendron*, as well as for the identification of functional genes, and the molecular regulatory networks of important traits. Another significant breakthrough has been achieved for gene functional identification in *Liriodendron*, with the transformation system having been established [9]. Based on this foundation, Dr. Xiaofei Long, in studying expression dynamics of *WOX* homeodomain transcription factors during somatic embryogenesis in *Liriodendron hybrids*, shed new light on potential functions of *WOX* genes during Liriodendron somatic embryogenesis [10]. In previous research, it was common to explore a gene’s function by overexpressing it in *Liriodendron*. The overexpression of the *LcPINa* gene has revealed that the PIN1 protein in *Liriodendron* plays a crucial role in the vegetative and reproductive growth of the plant. However, the overexpression of these genes can adversely affect the normal growth and development of the plant [11]. After strong promoter activation, the gene expression level deviated from its normal expression level, and the resulting phenotypic traits used to speculate on gene function were not completely accurate and comprehensive. Therefore, the DEX/GR inducible system was successfully established in *Liriodendron hybrids*, offering a valuable tool for the precise control and utilization of TFs at the desired levels [12]. The generation of traditional mutants relies on natural mutation, physical or chemical mutagenesis, and other methods [13]. Mutagenesis offers an alternative means of deriving insights into the gene function. Gene-editing technology enables targeted gene knockouts, which can provide new evidence for gene function research.

Carotenoids are one of the pigment types found in the leaves, stems, flowers, fruits, and other parts of plants. Phytoene desaturase (PDS) plays an important role in the formation of carotenoids. It is located upstream of the carotenoid synthesis pathway and can catalyze the dehydrogenation of colorless phytoene into colored carotenoids, first ζ-carotene, which in turn is converted into lycopene [14]. When the *PDS* gene is knocked out, carotenoids do not accumulate, photobleaching occurs, and the leaves have a visible phenotype [15]. Due to its visible phenotype, it is a useful target gene for establishing a gene-editing system. The use of the CRISPR/Cas system is widespread in herbaceous plants. Targeted genome-editing operations such as targeted knockout and insertion of multiple genes have been successfully performed in rice, wheat, Arabidopsis, and tobacco. In rice, laboratories have used CRISPR/Cas9 gene-editing technology to knock out the *OsPDS* gene, resulting in a homozygous mutant with a functional deletion of the *OsPDS* gene in the T0 generation. The mutant exhibits the expected albinism and dwarfism phenotype [16] and was highly evaluated by Science’s news focus article in 2013 [17]. In addition, Jen Sheen’s laboratory used the CRISPR/Cas system to achieve site-directed mutagenesis of the genome in Arabidopsis and Bunyan tobacco, with a mutation efficiency of up to 38.5%, and achieved site-directed editing of *AtRACK1b*, *AtRACK1c*, and *AtPDS3* [18]. Sophien Kamoun’s laboratory successfully utilized the CRISPR/Cas system to achieve site-directed mutation in the genome of native tobacco, with a mutation efficiency of 1.8% to 2.4% [19]. In addition, Zhu Jiankang and others used this system to conduct genome editing on Arabidopsis and rice, with a mutation rate of 84% at its most efficient [20]. In terms of woody plants, there is still relatively little research in this area. The CRISPR/Cas9 system is available only in several poplar species (e.g., *Populus tomentosa*, *Populus davidiana × Populus Bollena*) [21,22,23], Chinese pine [24], and several fruit trees (such as apple, banana, and grape) [25,26,27,28]. CRISPR/Cas system gene-editing technology has not yet been implemented in *Liriodendron*. Achieving biallelic/homozygous mutations in forest trees is a formidable challenge. Therefore, this study will use the transgenic methods established for *Liriodendron* to construct a CRISPR/Cas9 vector and carry out targeted knockout of the *LtPDS* gene in *Liriodendron*, providing a reference basis for research on and the application of the CRISPR/Cas system in woody plants. Gene editing in the callus tissue also provides new ideas for our mutant material preservation methods.

## 2. Results

### 2.1. L. tulipifera LtPDS Gene Cloning and Target Site Selection

**Figure 1:**
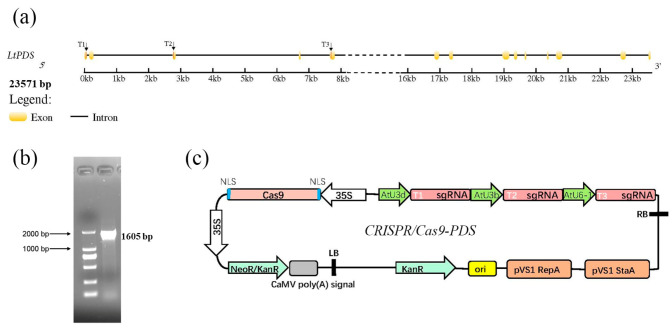
Location of the chosen CRISPR targets and schematic structure of the gene-editing vector used. (**a**) Structure of the *L. tulipifera LtPDS* gene. T1–T3 indicate the target locations. (**b**) PCR amplification of the *LtPDS* gene. (**c**) Schematic structure of the CRISPR/Cas9 gene-editing vector containing the T-DNA region.

The *AtPDS3* gene sequence was used to identify six homologous genes (LITU05G0617, LITU17G0003, LITU19G1614, LITU19G1621, LITU17G0001, and LITU17G0002) within the *L. tulipifera* genome. To verify the presence and activity of these six genes, we designed specific primers for gene cloning. Finally, only one gene was successfully cloned, named *LtPDS* (Figure 1a,b). The other five genes appeared to not be actively transcribed under our conditions.

We then analyzed the *LtPDS* gene sequence. We found that the total length of the genomic DNA sequence is 23,571 bp, including 14 exons and 13 introns. The total exon length is 1605 bp. A small number of SNP sites exist between two alleles (Figure S1). Based on targeting specificity, we designed three target sites for each vector (T1, T2, and T3). We designed two sets of target sites for the P33Fn vector, named *CRISPR/Cpf1-PDS1* and *CRISPR/Cpf1-PDS2* (Figure S2a,b). In addition, we constructed vector *CRISPR/Cas9-PDS* based on pYLCRISPR/Cas9P35S-N containing three sgRNA expression cassettes, in which T1-sgRNA, T2-sgRNA, and T3-sgRNA were expressed under the control of AtU3d, AtU3b, and AtU6-1, respectively (Figure 1a,c).

### 2.2. Comparative Analysis of Phenotypes in Different CRISPR Systems for Transient Transformation

**Figure 2:**
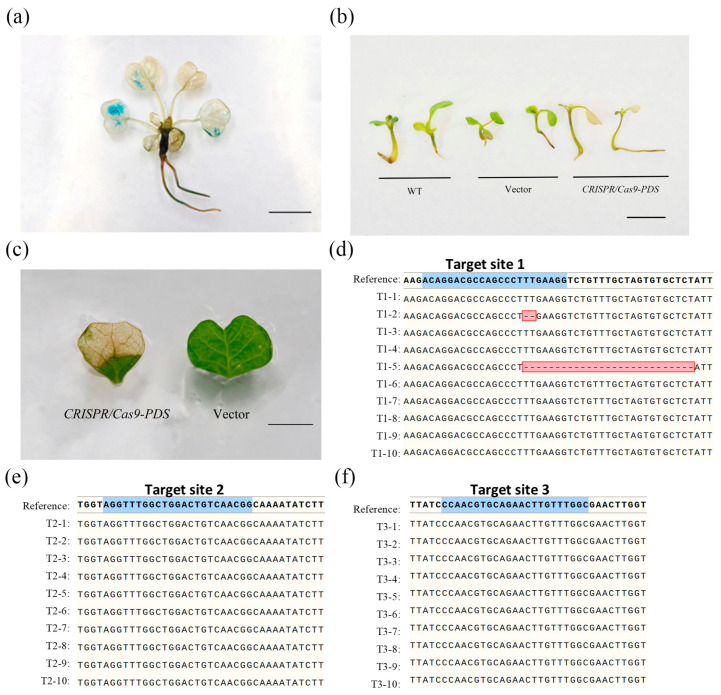
Transient genetic transformation of the CRISPR/Cas9 system. (**a**) Liriodendron seedlings after transient transformation of p35S:GUS. Scale bars, 1 cm. (**b**) Liriodendron seedlings after transient transformation of the CRISPR/Cas9 system. Scale bars, 2 cm. (**c**) Locally bleached Liriodendron leaves after transient transformation of the CRISPR/Cas9 system. Scale bars, 0.5 cm. (**d**–**f**) Mutation status of bleached leaves at different target sites.

To swiftly ascertain the practicality and efficacy of the gene-editing system in *Liriodendron*, we transferred *CRISPR/Cpf1-PDS1*, *CRISPR/Cpf1-PDS2*, *CRISPR/Cas9-PDS*, and their corresponding empty vector into 45 DAI *Liriodendron* seedlings. In addition, we transferred *p35S:GUS* as a control. After 72 h, GUS staining was performed on the *p35S:GUS* transformed seedlings, and the staining results indicated that tissues with transient expression were concentrated in the leaves (Figure 2a). Approximately one week post-transformation with *CRISPR/Cas9-PDS*, we observed localized areas of albifaction on a subset of seedling leaves (Figure 2b,c). We excised these affected leaf tissues for DNA extraction and subsequent sequencing analysis. The sequencing data revealed gene editing only at T1, albeit with a notably low efficiency of 20% (Figure 2d–f). Interestingly, in the cases of both *CRISPR/Cpf1-PDS1* and *CRISPR/Cpf1-PDS2* transformations, we failed to detect any albifaction on the leaves, and the sequencing outcomes did not indicate any gene-editing events (Figure S3). Based on these findings, we hypothesize that the Cpf1 protein may not be functional, while Cas9 is functioning in *Liriodendron*.

### 2.3. Phenotype of CRISPR/Cas9 LtPDS-Targeted Liriodendron for Stable Transformation

**Figure 3:**
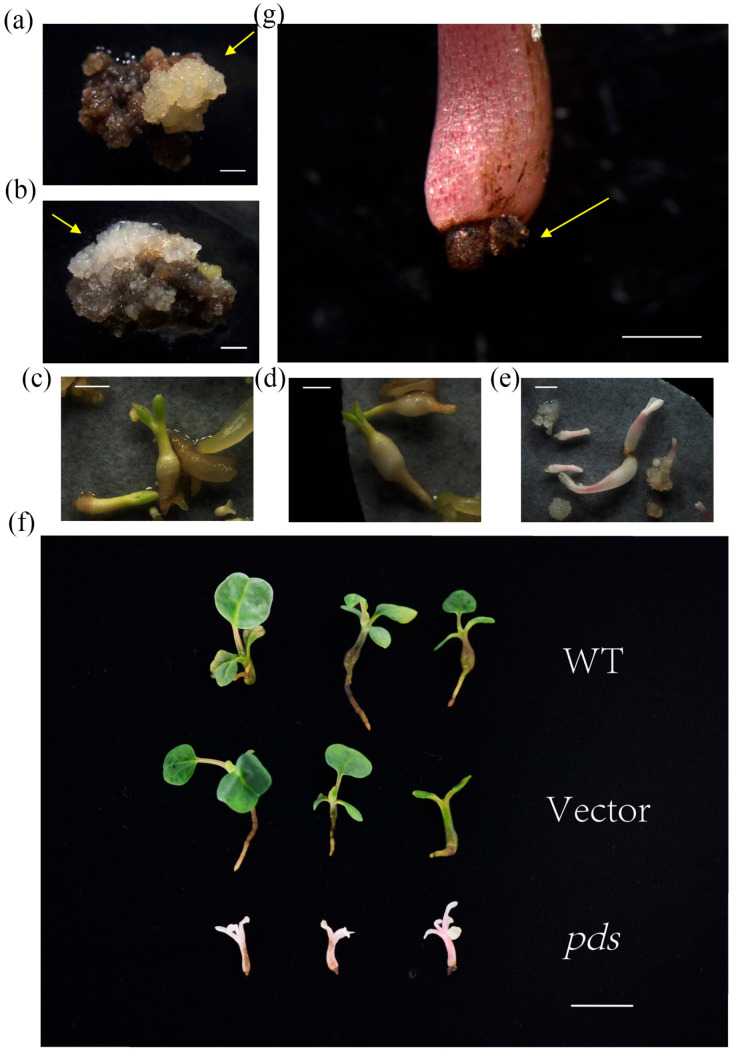
*L. tulipifera* phenotypes after stable genetic transformation with the CRISPR/Cas9 system. (**a**,**b**) Empty vector (**a**) and CRISPR/Cas9-PDS (**b**) callus during recovery culture after transformation. The yellow arrow indicates a callus emerging from the browning and a necrotic callus previously transfected with *Agrobacterium*. Scale bars, 2 mm. (**c**–**e**) Regeneration of plants from the embryogenic callus from the wild type (**c**), empty vector (**b**), and *pds* (**e**) via somatic embryogenesis after one month of dark cultivation and one week of light cultivation. Scale bar, 2 mm. (**f**) Representative phenotypes of wild type, empty vector, and *pds* seedlings after one month of illumination in SEM. Scale bars, 1 cm. (**g**) Roots of *pds* plants showing arrested root development. Scale bars, 1 mm.

After successfully achieving gene editing in *Liriodendron* through transient transformation, we aimed to develop a more efficient and stably heritable transformation method. Therefore, embryogenic callus of *L. tulipifera* was used as the receptor material for *Agrobacterium*-mediated transformation. Next, *L. tulipifera* calli infected by Agrobacterium were cultured on the MS medium for one week and then transferred into the selection medium for another two months. In that time, fresh calli began to emerge from the browning calli (Figure 3a,b). At this stage, we noticed that the fresh callus tissue transformed with CRISPR/Cas9-PDS exhibited a white appearance, whereas the tissue transformed with CRISPR/Cpf1-PDS1 and CRISPR/Cpf1-PDS2 displayed a yellow hue similar to the empty vector (Figure S4). After DNA extraction and PCR verification, we confirmed the success of transformation. We defined each small lump of new positive callus of CRISPR/Cas9-PDS as a line, obtaining a total of seven independent lines. The somatic embryos regenerated from dark culture were transferred to 3/4 MS medium after 7 days of illumination (Figure 3c–e). We found that CRISPR/Cas9-PDS transgenic seedlings showed a completely albino phenotype, while both empty vector and wild-type (WT) seedlings remained green (Figure 3f). We finally obtained a total of 137 regeneration plantlets, of which 113 (82.48%) exhibited an albino phenotype. We further compared the roots, stems, and leaves of seedlings. The leaves of albino seedlings all appeared white, developing slowly and with an irregular shape, while the leaves of WT and vector-transfected plants developed normally and remained green. The stems of albino seedlings appeared pink, and their roots atrophied, remained underdeveloped and turned brown (Figure 3g), while the stems of WT and empty vector seedlings were healthy and dark green. The stems of several WT seedlings were swollen at the base, the roots developed normally, and the root tips were white (Figure 3f). The albino plants were weak and small, eventually dying during cultivation.

### 2.4. Characterization of LtPDS Gene-Targeted Edits

By designing different primer pairs for each target site (Table S1), we amplified DNA segments spanning the three target sites (T1, T2, and T3) in the successfully transfected callus. Each PCR fragment was linked and transformed, and 10 single colonies were selected for Sanger sequencing. According to the statistical data of our sequencing results, among all the CRISPR/Cpf1-PDS1/2 callus tissues obtained, gene editing did not occur at any of the three target sites (Figure S5). Meanwhile, in seven lines (lines 1~7) obtained from the CRISPR/Cas9-PDS callus, mutations were found at the T1 site, with the highest mutation efficiency 20–100%. Some lines also had mutations at T2 (lines 1, 2, and 4, 30–60%), while no mutations were found at T3 (Table 1).

**Figure 4:**
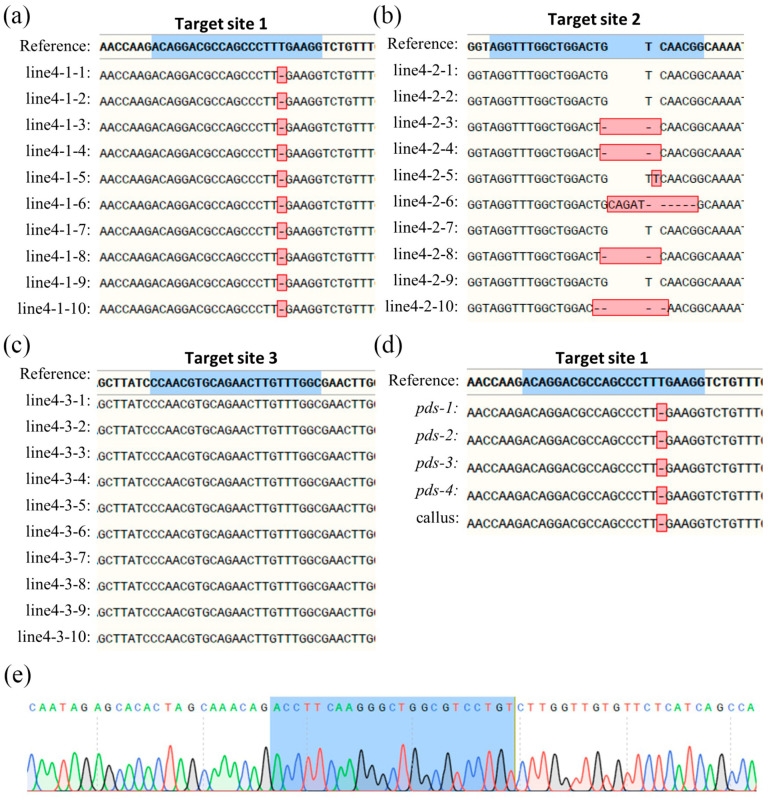
Sanger sequencing results of LtPDS gene target sites. (**a**–**c**) Mutation status of callus line 4 at different target sites. The red box represents the presence of mutations, and the blue box represents the designed target site. (**d**) Mutation of different plants at target site 1, where the callus is the calli tissue induced by a single mutant seedling. (**e**) One of the peak plots shown in panel d, where the blue box represents the designed target site.

We analyzed the types of mutations occurring at each target site (Figure 4a–c). We found that all mutations at T1 were simple deletions of 1 bp, which all occurred at the same location within the gene (Figure 4a). The mutations at T2 were simple deletions of 1-4 bp, simple insertions of 1 bp, and deletions accompanied by insertions (Figure 4b). No mutations were detected at T3 (Figure 4c).

Though Sanger sequencing on T1 of four random albino plantlets (pds-1/2/3/4), we found that only one mutation type, 1 bp deletion, was identified (Figure 4d). From the sequencing peak map, we found no overlapping peak (Figure 4e), which is preliminarily proposed to prove that the gene-editing type at this time is homozygous editing. In addition, Sanger sequencing on T1 of the callus (named callus), which was induced from one albino plantlet, showed the same mutation type (Figure 4d). We further used three primer pairs, Overlap-T1-F/R, Overlap-T2-F/R, and Overlap-T3-F/R, to amplify the DNA sequences of the three target sites (T1, T2, and T3) for these four albino plantlets. Fast NGS sequencing showed that the mutation at T1 of the PDS gene caused by CRISPR/Cas9 was homozygous, whereas heterozygous or chimeric knockout mutations were found at T2 (Table 2). Neither Sanger sequencing nor Fast NGS sequencing revealed mutations at the T3 site.

The high gene-editing efficiency of mutant plants pds-1/2/3/4 may be due to the fact that each plant develops from a single cell during somatic embryogenesis. The Fast NGS sequencing results of the callus showed the highest gene-editing efficiency at T1, and their editing types were consistent both at the T1 and T2 sites (Table 2). To further substantiate this point, we randomly selected albino seedlings induced to form a callus through dedifferentiation and conducted sequencing analysis on them. The results showed an editing efficiency of 98.44% at the T1 site, with a consistent editing type, confirming it as biallelic/homozygous gene editing. This provides a practical method for our future research: after obtaining mutant plants with ideal phenotypes, we can dedifferentiate them again to obtain callus tissue for preservation. At this point, the callus tissue we obtained not only has a high mutation rate but also has a consistent editing type.

As such, we determined that regardless of the genetic transformation method, Cpf1 does not work in Liriodendron, and using embryogenic callus tissue as the recipient material and the CRISPR/Cas9 vector for stable genetic transformation is the combination with the highest gene-editing efficiency.

## 3. Materials and Methods

### 3.1. Plant Materials and Culture Conditions

In this research, we conducted self-pollination of *L. tulipifera* (Tennessee provenance) in 2019 at Longshan Forest Farm, Anji County, Zhejiang Province. Eight weeks after pollination, immature aggregated samaras were collected from the pollinated tree. Then, the seeds were stripped and surface-sterilized. The immature embryos were extruded on an ultraclean workbench and cultured on callus induction medium (CIM). Finally, the calli of a somatic line (genotype 19-TN-SP) were selected as the transgenic receptor material.

Plantlets were obtained from embryogenic calli based on our established *L. hybrid* somatic embryogenesis system [6]. The obtained positive calli were transferred to liquid M13 medium for a suspension culture for two weeks. The suspended cells were collected using sieves, transferred to embryo induction medium (EIM), and cultured at 23 °C in the dark. After one month of cultivation, plants were obtained that were then transferred to stem elongation medium (SEM) after one week of illumination. All media used in this study were prepared according to https://doi.org/10.3389/fpls.2022.802128 [9].

### 3.2. Cloning of the LtPDS Gene and Selection of Target Sites

The *LtPDS* gene sequence was predicted by comparing the known *AtPDS* gene (AT4G14210) sequence from Arabidopsis to the *L. tulipifera* genome using BLASTP (comparison threshold E value < 10^6^).

We selected a *L. tulipifera* seedling and extracted RNA using the FastPure Plant Total RNA Isolation Kit after it was frozen and ground in liquid nitrogen on an ultra-clean workbench. We then tested the RNA concentration using a Nanodrop2000 instrument and RNA integrity by applying gel electrophoresis. After the cDNA chain was obtained by reverse transcription using HiScript^®^ III 1st Strand cDNA Synthesis Kit, Vazyme Biotech (Nanjing, China) Co., Ltd., we designed primers according to the predicted *LtPDS* gene sequence (LITU05G0617-F, LITU05G0617-R) and cloned the *LtPDS* gene using Phanta^®^ Max Super Fidelity DNA Polymerase from Vazyme Biotech (Nanjing, China) Co., Ltd. The PCR products were purified using a TIANgel Purification Kit from TIANGEN BioTech (Beijing, China) Co., Ltd. and cloned and inserted into a pClone007 blunt vector, which was transformed into DH5α receptor cells. Finally, we obtained the correct sequence by Sanger sequencing in Sangon Biotech (Shanghai, China) Co., Ltd. All primer sequences are listed in Table S1.

All possible Cas9 target sites within the obtained sequence were identified with the online software CRISPR-Pv2.0 (http://crispr.hzau.edu.cn/CRISPR2/, accessed on 4 September 2021). Three target sites were selected based on their genomic location, GC content, and potential off-target scores.

### 3.3. Vector Construction

First, based on the principle of overlay extension PCR technology, we performed PCR on the vectors pYLsgRNA-AtU3d, pYLsgRNA-AtU3b, and pYLsgRNA-AtU6-1 using primers containing the target sites. During this process, three target sites were successfully cloned into the 5′ end of three gRNA sequences, respectively, completing the AtU3d-sgRNA1, AtU3b-sgRNA2, and AtU6-1-sgRNA3 expression cassettes.

Based on Gibson assembly technology, the constructed expression cassettes were sequentially primed with adaptor primers for second-round PCR. At this time, each expression cassette had complementary pairing sequences upstream and downstream, so multifragment recombination (Gibson Assembly Kit) could be performed to construct the final expression vector (pYLCRISPR/Cas9P_35S_-N). All primer sequences are listed in Supplementary Table S1. We used the TIANprep Midi Plasmid Kit to extract the plasmid and transfer it into EHA105 Agrobacterium. The original vectors and primer sequences were from Xingliang Ma and Yao-Guang Liu [29].

The crRNA expression cassette within the CRISPR/Cpf1 vector is synthesized by Sangon Biotech (Shanghai, China) Co., Ltd. Following this, primers equipped with adaptors are utilized for the PCR amplification of the expression cassette. The resulting PCR product then undergoes homologous recombination with the enzymatically digested p33Fn plasmid. For this recombination step, the ClonExpress^®^ II One Step Cloning Kit from Vazyme Biotech (Nanjing, China) Co., Ltd. is employed.

### 3.4. Transient Transformation of Liriodendron Seedlings

We used 45 Days After Illumination (DAI) *Liriodendron* seedlings as recipient materials for transient transformation. The transformed Agrobacterium was cultured to 0.8 OD600, and the bacteria were collected and resuspended in 50 mL transformation solution. The seedlings were directly immersed in the transformation solution at 25 °C, 90 rpm for 3 h. The seedlings were then transferred to a washing solution for a quick 1 min rinse, and the bacterial solution was blotted dry with sterile paper towels. The seedlings were transferred to MS medium for cultivation and observation. The preparation methods for the transformation and washing solutions were referenced from https://doi.org/10.1007/s11103-017-0620-x [30]. The *p35S:GUS* used as a positive control originated from the *pCAMBIA 1301* vector.

### 3.5. Stable Transformation of Embryogenic Callus

We used our previously published *L. hybrid* transgenic system to transfer, via *Agrobacterium*, the constructed vectors and empty vectors into *L. tulipifera* callus. Approximately 50 mL of 0.6~0.8 OD600 bacterial solution was transferred into a 50 mL centrifuge tube, which was spun at 4 °C and 5000 rpm for 10 min, after which the supernatant was removed. An appropriate amount of M13 liquid medium was added to resuspend the sediment so that the OD600 of the resuspension reached ~0.8. On the ultra-clean worktable, 19-TN-SP calli were gathered into a conical flask, poured into the suspension, and co-cultivated for 10–15 min. Finally, the callus was collected and transferred to solid M13 medium (+Acetosyringone), after which it was cultured in the dark at 23 °C for 36–48 h.

After cleaning residual *Agrobacterium* on the callus with liquid M13 (+/− Cefotaxime) medium and ddH2O (+/− Cef), the calli were then collected again and transferred to solid M13 medium (+Cef) for a week of recovery culture, adding cefatoxime (+Cef). After one week of culture, the callus was transferred to solid M13 (+Geneticin+Cef) medium for screening and cultured until new positive calli grew from the original callus. The dosage of all reagents was sourced from https://doi.org/10.3389/fpls.2022.802128 [9].

### 3.6. Identification of Positive Lines and Detection of Mutations

After a week of recovery culture and approximately two months of screening culture, new calli began to appear on the browning and necrotic calli infected by *Agrobacterium*. We defined the calli expanded from each piece of new callus as a line. A small piece of callus was taken from each line for DNA extraction. The callus DNA was extracted using the CTAB method. After that, we designed primers of T1-F/R, T2-F/R, and T3-F/R (Supplementary Table S1) located 50–100 bp up- and downstream of the Target Site 1 (T1), T2, and T3 sequences within the genome and used Phanta^®^ Max Super-Fidelity DNA Polymerase to PCR amplify these stretches of DNA. The PCR product was directly cloned and inserted into a pClone007 blunt vector and transformed into DH5α *Escherichia coli*. Ten monoclonal colonies of each sample were sent to Tsingke Biotech (Nanjing, China) Co., Ltd., for sequencing. The mutation sites can be observed by comparing the sequencing results with the genome sequence.

The same method was used to detect mutations in the genomic DNA of regenerated plants. DNA was extracted using the CTAB method. After confirming that the vector was successfully transferred by PCR, PCR amplification was carried out for each target site using a pair of primers (Overlap-T1-F/R, Overlap-T2-F/R, and Overlap-T3-F/R) with adaptors (the adaptor sequence was provided by Tsingke Biotech (Nanjing, China) Co., Ltd.). The amplification products were sent to Tsingke Biotech (Nanjing, China) Co., Ltd., for Fast NGS sequencing.

## 4. Discussion

### 4.1. The Application of Different Genetic Transformation Systems in the Genetic Editing of Woody Plants

This study represents the first successful application of the CRISPR/Cas9 system in *Liriodendron*. We found that despite the novelty of the application, the highest mutation efficiency among the currently obtained mutant lines was 100% (Table 1). In other woody plants, for example, in *Populus tomentosa*, the primary gene-editing efficiency obtained was 51.7% [21]. The first application of CRISPR/Cas9 in *Citrus sinensis* cv. Valencia showed a gene-editing efficiency of 3.2–3.9% [31]; 0.1–6.9% was observed in *Malus prunifolia* cv. Golden Delicious, and 31.8% was observed in *M. prunifolia* (Wild.) Borkh. ‘Seishi’ × *M. pumila* Mill. var. *paradisiaca* Schneid. ‘M.9’ [25]; 3.74% to 20.11% was observed in *Hevea brasiliensis* [32]; and an efficiency of 97.1–98.9% was observed in *Manihot esculenta* cv. 60444 [33]. We found that when PEG-mediated genetic transformation was used, the efficiency was generally lower (*M. prunifolia* cv. Golden Delicious, *H. brasiliensis*), while the efficiency of Agrobacterium-mediated genetic transformation was generally higher (*M. prunifolia* (Wild.) Borkh. ‘Seishi’ × *M. pumila Mill*. var. paradisiaca Schneid. ‘M.9’, *Manihot esculenta*). Acceptor materials also play an important role in this process. When protoplasts were used as acceptor materials, the transformation efficiency was the lowest (*M. prunifolia*, *Vitis vinifera* L. cv. Chardonnay) [34]; in leaf discs, it was noticeably higher (*P. tomentosa*, *Actinidia chinensis*) [35]; and the highest transformation efficiency was found in the callus (*Dendrocalamus latiflorus* Munro, *V. vinifera* cv. Thompson, and *M. esculenta*) [28,36]. In this study, stable genetic transformation mediated by *Agrobacterium tumefaciens* was used, and the recipient material was an embryogenic callus, resulting in a very high transformation efficiency.

In addition, high genomic heterozygosity in woody plants has always been a major difficulty to overcome in the application of gene editing. The recipient material (19-TN-SP callus) selected in this study was induced from seeds produced by a self-pollinating *L. tulipifera* variety from the Tennessee provenance. The sequencing results showed that there were just two SNP sites present in the target-site sequences designed for the *PDS* gene. Therefore, there appears to be a low chance for target failure due to the presence of SNPs in this variety.

### 4.2. Utilizing the Somatic Embryogenesis System Based on a Single-Cell Origin for Gene Editing Can Result in Homozygous Mutants

Existing research indicates that the somatic embryogenesis system is highly likely to originate from a single cell [7,8]. The single-cell-origin somatic embryogenesis system is of significant importance for gene editing. Gene editing at the single-cell stage can improve the transformation efficiency because the transformed cells can directly participate in the embryo development process, rather than attempting to integrate into already formed tissues at a later stage. During the gene-editing process, if editing occurs at the multicellular stage, it may lead to the formation of chimeras, meaning that there are cells with different genotypes within the same plant. The somatic embryogenesis system based on a single-cell origin can reduce the formation of such chimeras because editing is performed in a single cell, which then develops into a complete plant. Gene editing at the single-cell level can enhance the precision of editing, as it allows for precise modifications to the gene before cell division and differentiation, ensuring that all progeny cells contain the same edits. Using the somatic embryogenesis system, a large number of genetically uniform plants can be rapidly regenerated from a single cell that has undergone gene editing, thus accelerating the breeding process.

In this research, we achieved a gene-editing efficiency close to 100%. And in the *pds* seedlings obtained in this study, there was no phenotypic differentiation in their appearance, as was previously found in poplar [23]. All seedlings showed a consistent albino phenotype. We speculated that all mutant lines contained biallelic mutations. The results that we obtained from Fast NGS sequencing confirm this point. The callus induced directly from mutant plants similarly showed the presence of biallelic mutations. The single-cell origin of somatic embryogenesis may be the reason for the high efficiency of homozygous editing, and it is also an effective pathway for future gene editing, especially for perennial, highly heterozygous woody plants. We artificially created homozygous recipient materials, which is of great significance for the rapid verification of the gene function and the conduct of gene editing. It is not necessary to wait for the long sexual generation to obtain regenerated plants with homozygous edits.

Another important reason for the high efficiency of homozygous editing is that there is only one *LtPDS* gene (LITU05G0617) in *Liriodendron*, with no other homologous gene present. Although six homologous genes were initially predicted, subsequent analysis revealed that none of the other five genes are actively transcribed and are therefore likely to be pseudogenes. Efficient biallelic mutations are closely related to the same sequence of the target allele. Therefore, a target sgRNA can simultaneously target two alleles, and the obtained mutant plant exhibits a homozygous albino phenotype. *PDS* encodes an enzyme named phytoene desaturase, which catalyzes the conversion of phytoene to phytofluene in the carotenoid biosynthesis pathway. It is located upstream of the carotenoid synthesis pathway and can catalyze the dehydrogenation of colorless phytoene into colored carotenoids, first ζ-carotene, which in turn is converted into lycopene [14]. This pathway is essential for the production of carotenoids, which are important for photosynthesis and photoprotection. When the *LtPDS* gene is knocked out, it results in a deficiency in carotenoid production, which manifests as the characteristic albino and dwarf phenotypes observed in *pds* mutants. The phenotypes of *pds* mutants are very consistent and obvious across all plant species in which they have been identified.

### 4.3. Gene-Editing Vector Optimization

In *Liriodendron*, we have made multiple attempts to employ the Cpf1 protein for gene editing, and yet we have not been able to successfully achieve gene editing. When we used the CRISPR/Cas9 system, the efficiency of gene editing was significantly enhanced. The CRISPR/Cas9 vector used in this study was previously published by Liu Yaoguang [29]. Three targets were designed in the present study, each linked to the same sgRNA sequence and regulated by three different promoters, AtU3d, AtU3b, and AtU6-1. It has been reported that the efficiency of the AtU3 promoter is much higher than that of AtU6 [37]. This is consistent with the results of this study (Table 1). The Pol III promoter sequences used in this study were all derived from Arabidopsis. We speculated that if the U3/U6 promoter of Liriodendron could be used to regulate the expression of the expression cassettes, it may lead to a higher editing efficiency. In addition, we used a 35S Pol II promoter to drive the expression of Cas9. However, there are a number of commonly used efficient promoters, such as ubiquitin, whose application efficiency in Liriodendron is unknown and should therefore be tested in the future.

## 5. Conclusions

We first established and optimized a highly efficient multi-target editing system in the magnoliid woody plant *Liriodendron tulipifera* via the CRISPR/Cas9 system and successfully obtained homozygous mutant plants based on single-cell-originated somatic embryogenesis.

## Figures and Tables

**Table 1:** Statistics of mutation efficiency at different target sites in each callus line.

Transgenic Lines	Mutation Efficiency		
	T1	T2	T3
1	100%	30%	0%
2	100%	40%	0%
3	30%	0%	0%
4	100%	60%	0%
5	20%	0%	0%
6	40%	0%	0%
7	60%	0%	0%

**Table 2:** Target site mutation as identified by Fast NGS sequencing.

	Target Site	Sequence	Mutation Type	Number of Reads	Mutation Proportion	Genome-Editing Type
*pds-1*	T1	ACAGGACGCCAGCCCTTTGA**AGG**	-	311	1.73%	homozygous
		ACAGGACGCCAGCCCTT------	Deletion	17,554	97.43%	
	T2	AGGTTTGGCTGGACTGTCAA**CGG**	-	2079	15.03%	chimeric
		AGGTTTGGCTGGACT--------	Deletion	8590	62.09%	
		AGGTTTGGCTGGAC---------	Deletion	2955	21.36%	
	T3	GCCAAACAAGTTCTGCACGT**TGG**	-	5732	100%	no mutation
*pds-2*	T1	ACAGGACGCCAGCCCTTTGA**AGG**	-	1595	9.88%	homozygous
		ACAGGACGCCAGCCCTT------	Deletion	14,374	89.05%	
	T2	AGGTTTGGCTGGACTGTCAA**CGG**	-	5432	49.64%	heterozygous
		AGGTTTGGCTGGACT--------	Deletion	4576	41.82%	
		AGGTTTGGCTGGACTGT------	Deletion	311	2.84%	
		AGGTTTGGCTGGACTG-------	Deletion	276	2.52%	
		AGGTTTGGCTGGACTGGCAACGG	Mismatch	140	1.28%	
	T3	GCCAAACAAGTTCTGCACGT**TGG**	-	7764	100%	no mutation
*pds-3*	T1	ACAGGACGCCAGCCCTTTGA**AGG**	-	895	5.01%	homozygous
		ACAGGACGCCAGCCCTT------	Deletion	16,786	93.98%	
	T2	AGGTTTGGCTGGACTGTCAA**CGG**	-	5277	40.36%	chimeric
		AGGTTTGGCTGGACT--------	Deletion	4666	35.69%	
		AGGTTTGGCTGGACTG-------	Deletion	2669	20.41%	
		AGGTTTGGCTGGACTGT------	Deletion	279	2.13%	
	T3	GCCAAACAAGTTCTGCACGT**TGG**	-	7356	100%	no mutation
*pds-4*	T1	ACAGGACGCCAGCCCTTTGA**AGG**	-	929	7.08%	homozygous
		ACAGGACGCCAGCCCTT------	Deletion	12,076	91.97%	
	T2	AGGTTTGGCTGGACTGTCAA**CGG**	-	5406	37.76%	chimeric
		AGGTTTGGCTGGACT--------	Deletion	5058	35.33%	
		AGGTTTGGCTGGACTG-------	Deletion	3429	23.95%	
		AGGTTTGGCTGGACTGT------	Deletion	231	1.61%	
	T3	GCCAAACAAGTTCTGCACGT**TGG**	-	7274	100%	no mutation
callus	T1	ACAGGACGCCAGCCCTTTGA**AGG**	-	163	0.92%	homozygous
		ACAGGACGCCAGCCCTT------	Deletion	17,371	98.44%	
	T2	AGGTTTGGCTGGACTGTCAA**CGG**	-	11,759	74.94%	heterozygous
		AGGTTTGGCTGGACTG-------	Deletion	3785	24.12%	
	T3	GCCAAACAAGTTCTGCACGT**TGG**	-	8048	100%	no mutation

Bold represents PAM sites.

## Data Availability

The original contributions presented in this study are included in the article/Supplementary Material, and further inquiries can be directed to the corresponding author.

## Supplementary Materials

The following supporting information can be downloaded at: https://www.mdpi.com/article/10.3390/plants14030472/s1, Figure S1: Alignment of cDNA sequences of two *LtPDS* alleles; Figure S2: Location of the chosen CRISPR targets and schematic structure of the gene-editing vector used; Figure S3: Transient genetic transformation of the CRISPR/Cpf1 system; Figure S4: Liriodendron callus during the recovery culture after stable transformation of the CRISPR/Cpf1 system; Figure S5: Sanger sequencing results of the CRISPR/Cpf1 system at *LtPDS* gene target sites; Table S1: Summary of all primer sequences.
