# Supplementary material for: Highly Efficient Homozygous CRISPR/Cas9 Gene Editing Based on Single-Cell-Originated Somatic Embryogenesis in Liriodendron tulipifera
Source: Plants (Basel). 2025 Feb 5;14(3):472. doi: 10.3390/plants14030472 (PMC11820044; doi:10.3390/plants14030472)
Supplement: Supplementary file 1 [file plants-14-00472-s001.zip › plants-3450505-supplementary.pdf]

Supplementary Material

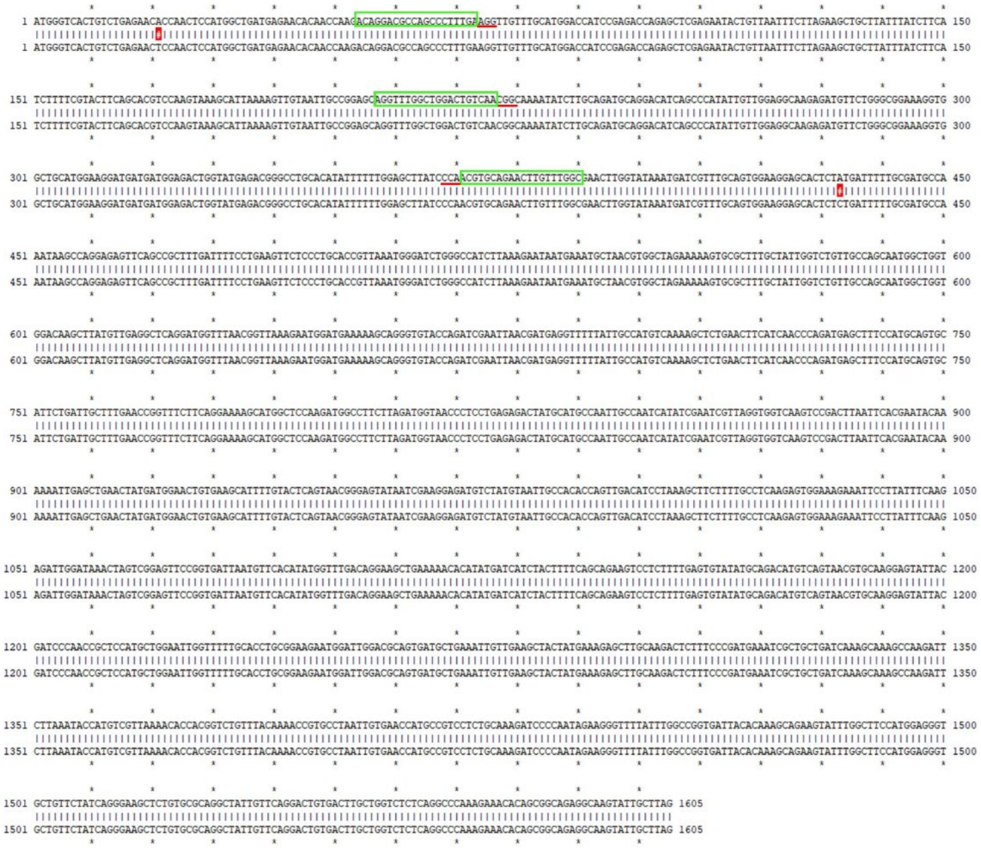

Figure S1. Alignment of cDNA sequences of two *LtPDS* alleles. The red '#' is the SNP site. The green boxes show three target sites, and the red lines show PMC sites in *CRISPR/Cas9-PDS* vector. The third target site was designed on the basis of the complementary chain.

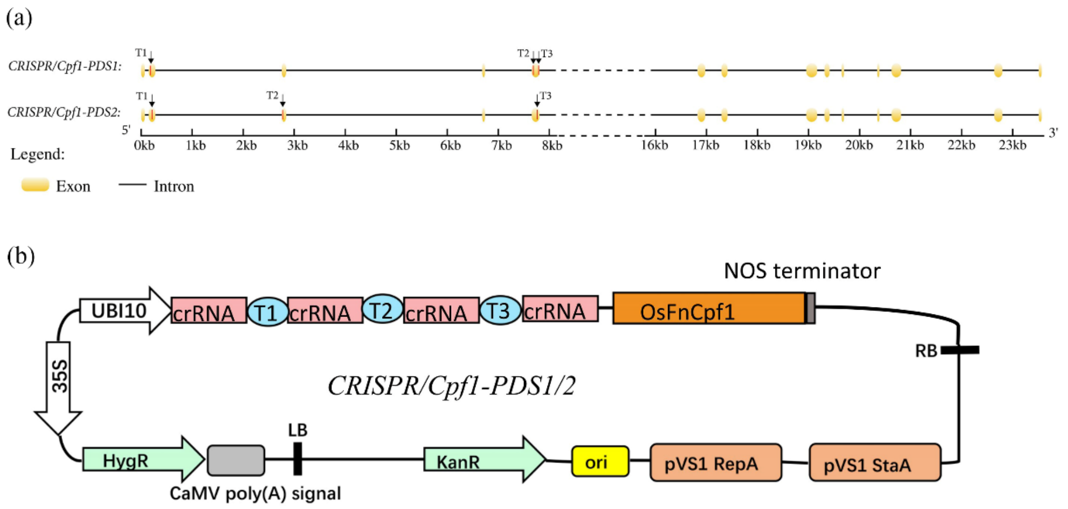

Figure S2. Location of the chosen CRISPR targets and schematic structure of the gene editing vector used. (**a**) Structure of the *L. tulipifera LtPDS* gene. T1-T3 indicate the target locations. (**b**) Schematic structure of the *CRISPR*/*Cpf1-PDS1*/*2* gene editing vector containing the T-DNA region.

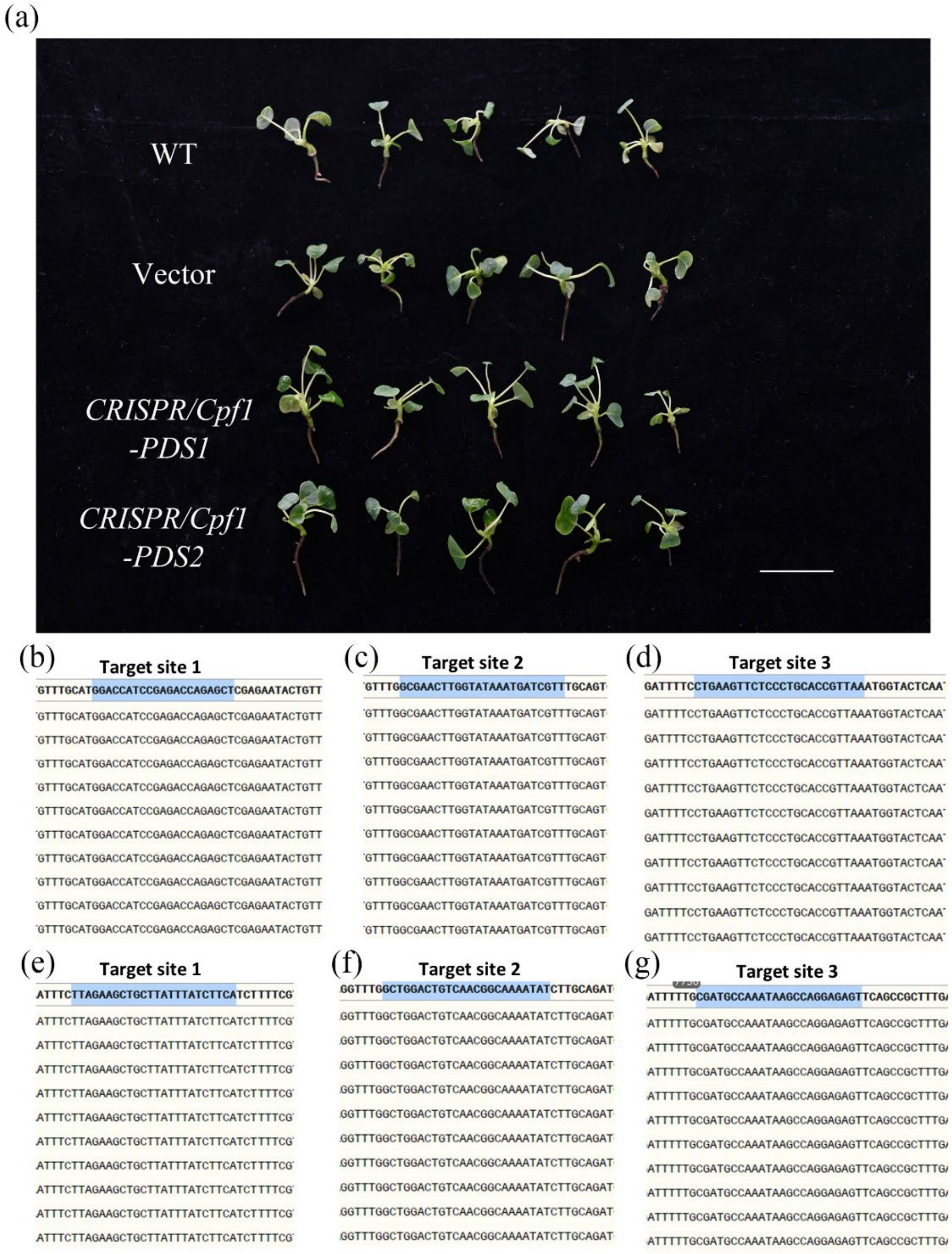

Figure S3. Transient genetic transformation of the *CRISPR/Cpf1* system. (**a**) *Liriodendron* seedlings after transient transformation of *CRISPR/Cpf1-PDS1/2*. Scale bars, 2 cm. (**b-d**) Mutation status of *Liriodendron* seedlings after transient transformation of *CRISPR/Cpf1-PDS1* at different target sites. (**e-g**) Mutation status of *Liriodendron* seedlings after transient transformation of *CRISPR/Cpf1-PDS2* at different target sites.

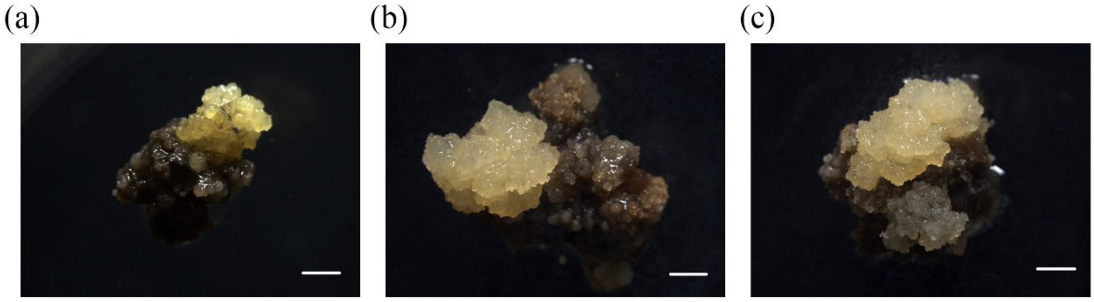

Figure S4. *Liriodendron* callus during the recovery culture after stable transformation of the CRISPR/Cpf1 system. (A) Callus transformed with empty vector. (B) Callus transformed with *CRISPR/Cpf1-PDS1*. (C) Callus transformed with *CRISPR/Cpf1-PDS2*. Scale bars, 2 mm.

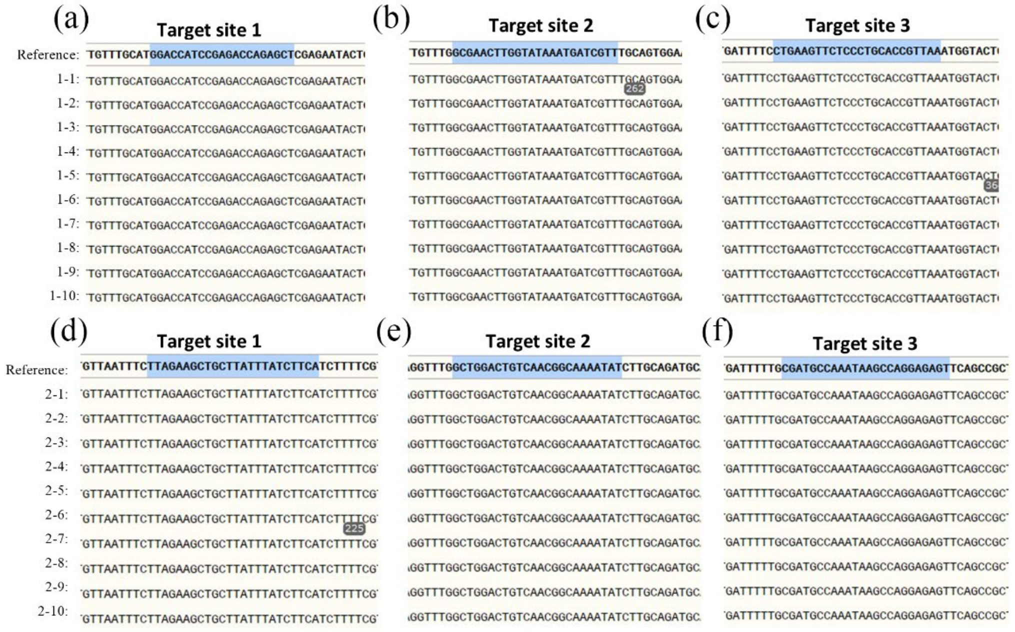

Figure S5. Sanger sequencing results of the CRISPR/Cpf1 system at *LtPDS* gene target sites. (**a-c**) Mutation status of callus after stable transformation of *CRISPR/Cpf1-PDS1* at different target sites. (**d-f**) Mutation status of callus after stable transformation of *CRISPR/Cpf1-PDS2* at different target sites.

Table S1. Summary of all primer sequences

Primer Name	Sequence 5'---3'
Primers of gene cloning	
LITU05G0617-F	ATGTAATTCACATCCCATTCAAAGTTG
LITU05G0617-R	CTTCTTTTGAACGCCTGATGATCG
Primers of expression cassettes construction	
U-F	CTCCGTTTTACCTGTGGAATCG
AtU3d-R	TCAAAGGGCTGGCGTCCTGTgaccaatggtgctttg
AtU3b-R	TTGACAGTCCAGCCAAACCTgaccaatgttgctcc
AtU6-1-R	ACGTGCAGAACTTGTTTGGCaatcactacttcgtct
gRT1-F	CAGGACGCCAGCCCTTTGAgttttagagctagaaat
gRT2-F	GGTTTGGCTGGACTGTCAAgttttagagctagaaat
gRT3-F	CCAAACAAGTTCTGCACGTgttttagagctagaaat
gR-R	CGGAGGAAAATTCCATCCAC
General primers of the second round PCR	
U-GAL	ACCGGTAAGGCGCGCCGTAGTGCTCGACAGTATGGAATCGGCAGCAAAGG
Pgs-GA2	CAGGGAGCGGATAACAATTTCACACAGGCAATCCACTCCAAGCTCTTG
U-GA2	GTGCCTGTGTGAAATTGTTATCCGCTCCCTGGAATCGGCAGCAAAGG
Pgs-GA3	CCACGCATACGATTTAGGTGACACTATAGCGCATCCACTCCAAGCTCTTG
U-GA3	CGCTATAGTGTCACCTAAATCGTATGCGTTGGAATGGCAGCAAAGG
Pgs-GAR	TAGCTCGAGAGGCGCGCCAATGATACCGACGCGTATCCATCCACTCCAAGCTCTTG
General primers of validation vectors	
SP-L2-New	gtcgtgctccacatgttgACCGGTAA
Pgs-R	TAGCTCGAGAGGCGCGCCAATGATACCGACGCGT
Primers of Target site PCR	
T1-F	ATGGGTCACTGTCTGAGAACAC
T1-R	TATTCTCGAGCTCTGGTCTCGG
T2-F	GCGCGTTCTCTGAATGTACCA
T2-R	GCTACATGAATCCTTTTCTCTTCAGGTC
T3-F	GCTTGCCATATTTCTCGAAGATTTCAC
T3-R	GCTCTGACCACAGATCAGTAGG
Primers of Fast NGS	

Overlap-T1-F	TCGTCGGCAGCGTCAGATGTGTATAAGAGACAGATGGGTCACTGTCTGAGAACAC
Overlap-T1-R	GTCTCGTGGGCTCGGAGATGTGTATAAGAGACAGTATTCTCGAGCTCTGGTCTCGG
Overlap-T2-F	TCGTCGGCAGCGTCAGATGTGTATAAGAGACAGGCGCGTTCTCTGAATGTACCA
Overlap-T2-R	GTCTCGTGGGCTCGGAGATGTGTATAAGAGACAGGCTACATGAATCCTTTTCTCTTCAGGTC
Overlap-T3-F	TCGTCGGCAGCGTCAGATGTGTATAAGAGACAGGCTTGCCATATTTCTCGAAGATTTCAC
Overlap-T3-R	GTCTCGTGGGCTCGGAGATGTGTATAAGAGACAGGCTCTGACCACAGATCAGTAGG
